# Predictors of Oral Rehydration Therapy use among under-five children with diarrhea in Eastern Ethiopia: a community based case control study

**DOI:** 10.1186/1471-2458-12-1029

**Published:** 2012-11-24

**Authors:** Bezatu Mengistie, Yemane Berhane, Alemayehu Worku

**Affiliations:** 1 College of Health Sciences, Haramaya University, P.O. Box 1570, Harar, Ethiopia; 2 Addis Continental Institute of Public Health, Addis Ababa, Ethiopia; 3 School of Public Health, Addis Ababa University, Addis Ababa, Ethiopia

**Keywords:** Case control study, Diarrhea, Predictors, Oral rehydration therapy

## Abstract

**Background:**

Rehydration therapy is a critical intervention to save the lives of children during the episodes of diarrhea. However, millions of children die every year due to failure to replace fluid effectively. The objective of this study was to identify the predictors of Oral Rehydration Therapy use among under-five children with diarrhea.

**Method:**

A community based unmatched case control study was conducted in Kersa district, Eastern Ethiopia, in February, 2011. The cases were 241 under-five children with diarrhea in the preceding two weeks before the survey and who had received Oral Rehydration Therapy while the controls were 253 under-five children with diarrhea in the preceding two weeks before the survey and who had not received Oral Rehydration Therapy. The cases and the controls were compared to find out the factors that were associated with the utilization of Oral Rehydration Therapy.

**Result:**

The study revealed that caregivers’ previous experience of Oral Rehydration Therapy use (AOR = 4.05, 95% CI = 2.63–6.22), seeking advice or treatment from health facilities, (AOR = 3.25, 95% CI = 2.06–5.11) and knowledge of Oral Rehydration Therapy (AOR = 3.09, 95% CI = 1.97–4.85) were found to be the positive determinants of Oral Rehydration Therapy use. Perception of teething as a cause of diarrhea was negatively associated with the utilization of Oral rehydration Therapy (AOR = 0.61, 95% CI = 0.37–0.98).

**Conclusion:**

Health education should be strengthened on the benefit, preparation, early initiation of Oral Rehydration Therapy and the causes of diarrhea. Attention should be given to those who do not have previous experience of Oral Rehydration Therapy use and have less frequent contacts with the health facilities.

## Background

Diarrhea is the second leading cause of child morbidity and mortality, especially in the developing countries. It is estimated that there are 2.5 billion episodes and 1.5 million deaths annually in children under-five years of age. This accounts for 21% of all the deaths in developing countries and the number has remained unacceptably high [1-4]. Diarrhea kills young children more than Acquired Immuno Deficiency Syndrome (AIDS), malaria and measles combined. It also exposes children to secondary infection. In Ethiopia, diarrhea is the major killer of children and thus is a serious public health problem. An estimated 73,700 children under the age of five die each year due to diarrhea. This accounts for an estimated 20% of the deaths among children under- five years of age in the country [5-7].

Oral Rehydration Therapy (ORT) is a primary intervention for the management of diarrhea. It can be easily administered at home by the mothers/caregivers as soon as a diarrhea episode begins [8,9]. ORT is simple, inexpensive and the most effective way to treat dehydration and reduce diarrhea mortality. Its use has been widely advocated mainly by World Health Organization (WHO) [2,10-12].

However, despite the extensive efforts made to promote ORT for the last several decades, its utilization by rural communities has remained unsatisfactory [11,13]. A recent review of literature showed that only 39% of the children with diarrhea in developing countries receive ORT [5]. Some studies have found that maternal perception of the causes of diarrhea, perception of ORT usefulness, barriers regarding its preparation were associated with ORT use [14-16]. Other studies indicated the relationship of ORT use with maternal education, residence, income of the household and age of the patient [15,17-19].

In Ethiopia, the government has tried to provide this life saving treatment to the rural communities by deploying health extension workers (village health agents) in every kebele (smallest administrative unit which on average has 5000 population) in the country. However, only 39.7% of the under-five year children with diarrhea received any form of ORT [20]. Furthermore, there was no significant increase in the utilization of ORT in the last ten years, and this has created great concern among stakeholders in the country [20-22]. A similar finding was reported based on Demographic and health surveillance (DHS) data analysis in 34 countries in Sub-Saharan Africa and Asia [13]. Investigating the factors associated with the utilization of such a widely available and effective life saving intervention is of paramount importance. Thus, the objective of this study was to identify the predictors of Oral Rehydration Therapy use among under-five children at the community level.

## Methods

Unmatched case control study was employed to assess the predictors of ORT use among children under-five years of age. It was conducted in Kersa district, Eastern Ethiopia, in February, 2011. The study area was chosen by Haramaya University to serve as a demographic surveillance and health research center. The study site has twelve kebeles with 55,394 residents. The cases were under-five children with diarrhea in the preceding two weeks and who had received ORT, while the controls were under-five children with diarrhea in the preceding two weeks and who had not received ORT.

The sample size of the study was determined using a formula for case control study with the assumptions: proportion of caregivers educated (exposure) among control to be 33.7% [21]; and detecting 1.74 times higher educated caregivers among the cases, 95% confidence level, 80% power, case to control ratio of 1:1 and 10% non-response rate. Accordingly, 253 cases and 253 controls were required for the study.

The cases and the controls were identified by conducting a canvas survey of all the households in the demographic surveillance village. A total of 6674 children under the age of five-years were surveyed in two days, and 956 children with diarrhea in the preceding two weeks were identified. Of these children, 243 were cases and 713 were controls. All the cases were included in the study except two non-respondents. A sampling frame which enlists all the eligible study subjects was prepared for the controls. Simple random sampling technique was used to select the controls from the sampling frame. Children with persistent diarrhea were excluded from the study.

A pre-tested structured questionnaire was used for data collection. The questionnaire was evaluated by pediatricians and public health professionals before the data collection. The data were collected by trained and experienced data collectors who were serving the demographic and health research center. Three supervisors of the demographic surveillance and health research center supervised the data collection. The data collectors and supervisors were all fluent in the local language. The respondents were primarily the mothers of the eligible under-five children, but in their absence, the next caregivers were interviewed. To ensure the validity of the data, supervisors checked the filled in questionnaire on a daily basis for consistency and completeness. Five percent of the households were interviewed by the supervisors to check the consistency of the collected data.

The independent variables were the child’s age and sex, the number of children in the household, educational status, residence, wealth status, occupation and the age of the caregiver, family size, knowledge about ORT, previous use of ORT, access to Oral Rehydration Solution (ORS), the presence of ORS at home during the onset of diarrhea, health seeking behavior, perceived cause and recognizing the severity of diarrhea and dehydration.

Diarrhea was defined following WHO protocol as [23], the passage of three or more loose stools over 24 hours period reported by mothers/caregivers. ORT was defined as the provision of ORS, home recommended fluids, or increase fluids. The outcome variable, ORT use, was measured based on WHO and national diarrhea treatment guideline. A child under two years of age and who received half a cup (50–100 ml) of ORT or a two years of age and above and who received a cup (100–200 ml) of ORT after each loose stool was considered as ORT user [24,25]. Caregivers were asked the amount of ORT given in cup, as most were not familiar with metric measurement system.

Access to ORS was determined by inquiring the health facilities where ORS could be found and by estimating the time to walk to the nearest facility. Less than one hour walking distance from home to the health facilities was considered as “had access” and one hour or longer walking distance was considered as “no access”.

To assess their knowledge, caregivers were asked to respond to five questions. These were ever hearing, preparation, initiation, benefits of ORT, and when to use the prepared ORS. The responses were given 1 score for the correct and 0 for the incorrect. The values of the five variables were combined to produce a total knowledge score about ORT. Based on their responses, the knowledge of the caregivers was categorized as ‘good’ if the total score was greater than the mean score of the respondents and ‘poor’ if it was less than or equal to the mean score of the respondents. This categorization was made after the distribution of the data had been assessed. The caregivers’ socio-economic status was assessed using 28 selected variables. The variables used include income, occupation, ownership of assets, and ownership of farm land and other assets, bank savings, housing conditions and access to facilities such as sanitation, improved water supply. Using principal component analysis, the wealth status of the cases and the controls were leveled as poor, middle, or rich.

The data were double entered in to EpiData 3.1 by trained data clerks. SPSS version 16 software was used to analyze the data. Descriptive statistics was used to summarize the study variables. Unconditional logistic regression analysis with enter procedure was used to select the predictors and adjust confounding variables. To reduce excess number of variables in the final model, only those variables with P < 0.2 in the bivariate analysis were considered in the multivariate analysis, along with variables that were well known predictors such as maternal education and residence. Variables with P < 0.05 in the final logistic regression analysis were considered as significant. Multicollinearity of the independent variables was checked using Variance Inflation Factor (VIF), and these values (1.045–1.584) were below the recommended maximum VIF value of four [26].

Ethical clearance was obtained from the College of Health Science of Haramaya University. Informed written consent was obtained from each respondent after explaining the objective and procedure of the study. All responses were kept confidential.

## Result

A total of 241 cases and 253 controls were analyzed; the response rate was 97%. The mean (±SD) age of the cases and the controls was 24 (± 13.5) and 23.1 (± 12.9) months respectively. Nearly all 486 (98.4%) of the caregivers were biological mothers. The majority of the caregivers of the cases 202 (83.8%) and of the controls 223(88%) were illiterate (COR = 1.43, 95% CI = 0.86–2.39). Some of the caregivers of the cases 91 (37.7%) and of the controls 73 (28.8%) were in the rich category (COR = 1.65, 95% CI 1.06–2.55). Wealth status was positively associated with the use of ORT (Table 1).

**Table 1:** Socio-demographic and economic factors associated with ORT use among under-five children, Kersa district, Eastern Ethiopia, 2011

**Variables**	**Cases**	**Controls**	**Total**	**Crude OR (95%CI)**
Age of the child in months				
< 12	43 (17.8%)	53 (20.9%)	96	1
12–23	74 (30.7%)	69 (27.3%)	143	1.32 (0.78–2.22)
> = 24	124 (51.5%)	131 (51.8%)	255	1.16 (0.72–1.86)
Sex of the child				
Male	130 (53.9%)	122 (48.2%)	252	1
Female	111 (46.1%)	131 (51.8%)	242	1.25 (0.88–1.79)
Number of under-five children				
1	122 (50.6%)	123 (48.7%)	245	1
2	107 (44.4%)	109 (43.1%)	216	0.58 (0.27–1.24)
3	12 (5%)	21 (8.3%)	33	0.57 (0.27–1.22)
Residence				
Rural	207 (85.9%)	221 (87.4%)	428	1
Urban	34 (14.1%)	32 (12.6%)	66	1.13 (0.67–1.95)
Occupation of the caregiver				
Housewives	230 (95.4%)	247 (97.6%)	477	0.50 (0.18–1.39)
Other	11 (4.6%)	6 (2.4%)	17	1
Education of the caregiver				
Illiterate	202 (83.8%)	223 (88.1%)	425	1
Literate	39 (16.2%)	30 (11.9%)	69	1.43 (0.86–2.39)
Age of the caregiver				
<=30	180 (74.7%)	205 (81%)	385	1
>30	61 (25.3%)	48 (19%)	109	1.44 (0.94–2.22)
Family size				
<= 4 persons	76 (31.5%)	70 (27.7%)	146	0.83 (0.56–1.22)
>4 persons	165 (68.5%)	183 (72.3%)	348	1
Wealth status				
Poor	71 (29.5%)	94 (37.2%)	165	1
Middle	79 (32.8%)	86 (34%)	165	1.21 (0.78–1.87)
Rich	91 (37.7%)	73 (22.8%)	164	1.65 (1.06–2.55)

Knowledge about ORT was higher among the caregivers of the cases 139 (57.6%) compared to the controls 39(15.4%) (OR = 4.11, 95% CI = 2.8–6.02). From the total 323 caregivers who ever heard about ORT, the majority 255 (78.9%) had got the information from health workers. The majority of the caregivers of the cases 176 (73%) and few of the controls 98 (38.7%) had used ORT before (OR = 4.28, 95% CI = 2.97–6.26). Knowledge about ORT, accesses to ORS, previous use of ORT and seeking advice or treatment from the health facility were positively associated with ORT utilization (Table 2).

**Table 2:** Comparison of cases and controls according to caregiver behavior and access to ORS, Kersa district, Eastern Ethiopia, 2011

**Variable**	**Cases**	**Controls**	**Total**	**Crude OR (95%CI)**
Knowledge of ORT				
Good	139 (57.7%)	63 (24.9%)	202	4.11 (2.80–6.02)
Poor	102 (42.3%)	190 (75.1%)	292	1
Previous use of ORT				
Yes	176 (73%)	98 (38.7%)	274	4.28 (2.97–6.26)
No	65 (27%)	155 (61.3%)	220	1
Access to ORS				
Yes	87 (36.1%)	41 (16.2%)	128	2.92 (1.9–4.47)
No	154 (63.9%)	212 (83.8%)	369	1
Advice or treatment from health facilities				
Yes	117 (48.5%)	54 (21.3%)	171	3.47 (2.34–5.15)
No	124 (51.5%)	199 (78.7%)	323	1
Had ORS sachet at home				
Yes	24 (10%)	17 (6.7%)	41	1.53 (0.80–2.93)
No	217 (90%)	236 (93.3%)	453	1

Caregivers who perceived teething as the cause of diarrhea were less likely to provide ORT to their children than the caregivers who did not perceive teething as a cause of diarrhea (OR = 0.59, 95% CI = 0.41–0.87). Caregivers were asked about the signs of recognizing the severity of diarrhea such as fever, repeated vomiting, many watery stools, not able to eat or take fluid, marked thirst and blood in the stool. Twenty nine (12%) caregivers of the cases and 16(6.3%) caregivers of the controls identified two or more of these signs (OR = 1.74, 95% CI = 0.77–3.93). WHO recommended signs of dehydration to be identified by the mother or caregiver such as excessive thrust, sunken eye, reduced urine output, excessive drowsiness, poor skin turgor, restlessness, absent of tears were assessed. One hundred ninety three (80.1%) caregivers of the cases and 185(73.1%) caregivers of the controls identified only one of these signs correctly. Perceiving teething as a cause of diarrhea was negatively associated with ORT use (Table 3).

**Table 3:** Caregivers perceived causes of diarrhea and assessment of morbidity, Kersa district, Eastern Ethiopia, 2011

**Variable**		**Cases**	**Controls**	**Total**	**COR 95%CI**
Perceived causes of diarrhea					
Teething	Yes	67 (27.8%)	99 (39.1%)	166	0.59 (0.41–0.87)*
	No	174 (72.2%)	154 (60.9%)	328	1
Evil eye	Yes	17 (7.5%)	25 (9.9%)	42	0.69 (0.36–1.31)
	No	224 (92.9%)	228 (90.1%)	452	1
Infection/weaning	Yes	83 (34.4%)	59 (23.3%)	152	1.41 (0.93–2.05)
	No	158 (65.6%)	194 (76.7%)	352	1
No idea about the cause	Yes	74 (30.7%)	70 (27.7%)	144	1.15 (0.78–1.70)
	No	167 (69.3%)	183 (72.3%)	350	1
Number of signs identified to recognize the severity of diarrhea	none	27 (11.2%)	26 (10.3%)	53	1
	1	185 (76.8%)	211 (83.4%)	396	0.84 (0.47–1.49)
	2	29 (12%)	16 (6.3%)	45	1.74 (0.77–3.93)
Number of signs identified to recognize dehydration	none	34 (14.1%)	51(20.2%)	85	1
	1	193 (80.1%)	185 (73.1%)	378	1.56 (0.97–2.52)
	=>2	14 (5.8%)	17 (6.7%)	31	1.23 (0.53–2.83)
Type of diarrhea	Non- bloody	29 (12%)	24 (9.5%)	53	1
	bloody	212 (88%)	229 (90.5%)	441	1.13 (0.73–2.31)

Further multivariate analysis with logistic regression model was used to compute the adjusted OR and 95% confidence interval of each variable. In the first regression model, socio-demographic and economic variables (Residence, age of the caregiver, educational status and wealth status) were included. None of the four variables showed significant associations with ORT use (Table 4).

**Table 4:** Predictors of ORT use in the multivariate analysis, Kersa district, Eastern Ethiopia, 2011

**Variable**		**Model I OR (CI)**	**Model II OR (CI)**	**Final model OR (CI)**
Age of the caregiver	<=30	1		1
	>30	1.49 (0.96–2.30)		1.17 (0.70–1.95)
Educational status of the caregiver	Illiterate	1		1
	Literate	1.33 (0.66–2.11)		1.03 (0.53–2.03)
Residence	Urban	1.19 (0.47–1.49)		1.07 (0.49–2.33)
	Rural	1		1
Wealth index	Poor	1		1
	Middle	1.16 (0.75–1.81)		1.35 (0.80–2.27)
	Rich	1.60 (0.99–2.60)		1.43 (0.81–2.56)
Previous use of ORT	Yes		3.96 (2.59–5.81)**	4.05 (2.63–6.22)**
	No		1	1
Had ORS at home during diarrhea	Yes		1.71 (0.79–3.70)	1.81 (0.83–3.92)
	No		1	1
Seeking advice or treatment from HF	Yes		3.29 (2.11–5.15)**	3.25 (2.06–5.11)**
	No		1	1
knowledge of ORT	Good		3.13 (2.00–4.90)**	3.09 (1.97–4.85)**
	Poor		1	1
Perceived teething as cause of diarrhea	Yes		0.60 (0.38–0.97)*	0.61 (0.37–0.98)*
	No		1	1
Number of signs identified to recognize dehydration	none		1	1
	1		1.29 (0.73–2.28)	1.21 (0.67–2.20)
	=>2		0.95 (0.34–2.63)	0.94 (0.33–2.65)
Access to ORS	Yes		1.85 (1.11–3.09)	1.76 (0.97–2.98)
	No		1	1

* = p < 0.05; ** = p < 0.01; *HF*= health facility.

In the second regression model, the caregivers’ knowledge about ORT and the signs of dehydration, previous experience in using ORT, perceived causes of diarrhea, access to and possession of ORS, and advice or treatment seeking behavior were put together. Their previous experience in using ORT, knowledge about ORT, behavior of seeking advice or treatment from health facilities, and access to ORS were positively associated with the utilization of ORT, while teething as perceived cause of diarrhea was negatively associated (Table 4).

In the final model, variables from the first and the second model were put together. After controlling the effect of other variables seeking advice or treatment from health facilities (AOR = 3.25, 95% CI = 2.06–5.11), previous experience of ORT (AOR =4.05, 95% CI = 2.63–6.22), and Knowledge about ORT (AOR = 3.09, 95% CI = 1.97–4.85) were positively associated with ORT use. Teething as perceived cause of diarrhea was negatively associated with the provision of ORT (AOR = 0.61, 95% CI = 0.37–0.98) (Table 4).

## Discussion

We examined socio-demographic and care related characteristics associated with ORT use for the case management of diarrhea among children under five years old in Eastern Ethiopia. In this study the caregivers’ previous experience with of ORT use, seeking advice or treatment from health facilities and knowledge about ORT were associated with increased ORT use. Teething as perceived cause of diarrhea was associated with decreased ORT use.

In our study, previous use of ORT was positively associated with ORT use in the current episode of diarrhea. This finding is in agreement with studies done in Guniea – Bissau [14] and Nigeria [9], where caregivers who gave ORT for their children at any time in the past were more likely to use ORT during the current episode of diarrhea. This may be explained by the fact that familiarity with ORT could be gained through experience.

According to the Community Integrated Management of Childhood Illness (C-IMCI) strategy, caregivers at home should have adequate knowledge about the causes and the treatments of diarrhea [27]. In our study, the knowledge of the caregivers about ORT was positively associated with ORT use. A similar result was found in a study conducted in Dominica Republic [28]. Furthermore, the analysis of the 1992–2005 Demographic and health Surveillance (DHS) data from 34 countries indicated that one of the causes for the declining trend in compliance with ORT is the poor awareness of caregivers [13].

We found out that seeking advice or treatment from health facilities for the current episode of diarrhea was positively associated with ORT use. This finding is consistent with a study conducted in Kenya [17], where the caregivers who had sought care at the health facility were more likely to use ORT than their counterparts. In our study, health workers were information sources for the majority of the caregivers. In a qualitative study conducted in Kenya, many of the caregivers needed advice from the health workers to provide ORT to their children [29]. This is not in line with the WHO recommendation that fluids should be given immediately at the onset of diarrhea [23].

Parental false beliefs associated with teething may interfere with the prompt diagnosis and management of a range of serious illnesses [30]. In this study, teething as a perceived cause of diarrhea was negatively associated with ORT use. A similar study done in Nigeria [31] indicated that caregivers who perceived teething as a causes of diarrhea were less likely to use ORT for the case management of diarrhea than who did not. A qualitative study conducted in Malawi indicated that teething diarrhea was considered as a normal development of the child that needs no action or treatment [32]. This misconception has negative implications on the management of the disease.

In our study, the presence of ORS packet at home during the onset of diarrhea was not associated with ORT use. This is not in agreement with a study conducted in Guniea-Bissau [14]. Other study indicated that many caregivers are waiting the advice of the health workers to ensure that ORS is the suitable treatment [29]. This misunderstanding might be the reason for the lack of association between the presence of ORS at home and ORT use. But this needs further study in order to fully understand it.

In this study, caregivers’ educational status showed no difference between the two groups. A similar finding was observed in a study conducted in Nigeria [15]. However, this finding was inconsistent with other studies. Results of the studies in Vietnam [33], Nepal [34], India [35] and Czech [36] showed that a higher educational status of caregivers was found to be associated with a higher use of ORT among children under five.

There are limitations to this study. First, this study was based on the retrospective self reporting. Recall bias might have occurred. Secondly, the data collectors might have introduced bias during the interview but, since they were not aware of the hypothesis being tested, its effect is thought to be little. Thirdly, there could be social desirability bias in some of the responses such as seeking advice or treatment from health facilities that lead to overestimation. There are also strengths of the study. Cases and controls were identified by canvas survey before the interview, and this may reduce the selection bias. The controls were selected by simple random sampling technique. To minimize misclassification, we used the WHO guideline to define the cases.

## Conclusion

The study identified that the caregivers’ previous experience with ORT, knowledge about ORT, advice or treatment seeking behavior and perceived cause of diarrhea were the predictors of ORT use. This information could be very important to strengthen the strategies and the approaches to reach caregivers so as to increase the coverage of the ORT use. Health education should focus on the benefit, early initiation, and the preparation of ORT and the causes of diarrhea. It is also important to identify and encourage caregivers with no previous experience of ORT and who do not have frequent contact with the health facilities to use ORT at the onset of diarrhea. Further follow up studies that aim at observing the daily activities of the caregivers during the diarrhea episode are recommended.

## Competing interests

The authors declare that they have no competing interest.

## Authors’ contributions

BM, YB and AW participated from the conception to the final write up of the study. All authors read and approved the manuscript.

## Authors’ information

BM is assistant professor of public health at Haramaya University, Ethiopia. YB is a senior professor of epidemiology and public health and director of Addis Continental institute of public health, Ethiopia. He has been teaching several courses in public health including epidemiology and research methodology in various universities. He also has supervised many masters and doctoral students. He also has more than 100 publications in the national and international journals. AW is associate professor at Addis Ababa University, Ethiopia. He has been teaching biostatics and research methods in various universities for many years. He has more than 40 publications in the peer reviewed national and international journals.

## Pre-publication history

The pre-publication history for this paper can be accessed here:

http://www.biomedcentral.com/1471-2458/12/1029/prepub
